# Red eyes presenting with a fistulous lesion in the left lower extremity: a case report

**DOI:** 10.1186/s13256-015-0661-1

**Published:** 2015-09-11

**Authors:** Natalia Palmou Fontana, Enrique Judez Navarro, Oscar Martín Melero, Sergio Losa Palacios

**Affiliations:** 1 Rheumatology Department, Hospital General de Almansa, Av de la Circunvalacion s/n, (02640) Almansa, Albacete Spain; 2 Rheumatology department, Hospital del Perpetuo Socorro, Albacete, España; 3 Ophthalmology Department, Hospital General de Albacete, Albacete, España; 4 Traumatology Department, hospital General de Albacete, Albacete, España

## Abstract

**Introduction:**

Very few cases of scar sarcoidosis affecting the eyes and bone together have been reported in the last few years.

**Case presentation:**

We report a case of a 49-year-old Spanish man with recurrent bilateral granulomatous uveitis and a fistulous nodular lesion in the left pre-tibial region (scar granuloma) on the site of an 8-year-old scar. He presented with bilateral hilar adenopathies and elevation of inflammatory markers and angiotensin-converting enzyme. A histologically confirmed sarcoid of the tibia with a radiologic appearance unusual for long tubular bone involvement was observed. He also had bilateral ophthalmologic involvement.

**Conclusions:**

Sarcoidosis is a disease of unknown cause histologically characterized by non-caseating granulomas that can involve any organ or tissue. Osseous sarcoidosis is a relatively rare presentation. However, on the basis of cases reported in the literature, sarcoid lesions on bones are generally asymptomatic. Biologic agents are considered an alternative therapy for sarcoidosis resistant to conventional treatment.

## Introduction

Scar sarcoidosis of the tibia is considered a rare disease. It is usually described in old scars (10 years) [1], and it is more common in the upper extremities and facial area, together with an exacerbation of sarcoidosis symptoms (Löfgren syndrome) affecting the eyes or, in our patient, bone [2].

In the last few years, very few cases of scar sarcoidosis affecting the eyes or bone have been described in the literature. The appropriate treatment consists of steroids with gradual dose increases until the condition is controlled, followed by immunosuppressive agents such as methotrexate and azathioprine for control of inflammation [3]. Several studies support the use of monoclonal antibodies against tumor necrosis factor α, mostly infliximab, in the treatment of pulmonary sarcoidosis, uveitis, cutaneous sarcoidosis including lupus pernio, and neurosarcoidosis [4–12]. Nevertheless, evidence is based on small series or on case reports. We describe a case of a patient with recurrent bilateral granulomatous uveitis together with a fistulous nodular lesion in the left pre-tibial region (scar granuloma) on the site of an 8-year-old scar.

## Case presentation

A 49-year-old Spanish man was referred to our hospital by an ophthalmologist in June 2010 with a symptom of red eyes. A uveitis protocol was implemented. A chest X-ray was ordered. The X-ray showed mediastinal adenopathy, for which the patient was referred to the respiratory medicine department. The results of his respiratory function tests were normal, so a high-resolution computed tomographic (CT) scan of the lungs was ordered. As he had no interstitial disease, he was diagnosed with stage I pulmonary sarcoidosis, and no systemic treatment was prescribed. A sacroiliac X-ray and a magnetic resonance imaging scan showed sclerotic changes that appeared chronic. No bone edema was observed. His human leukocyte antigen B27 test was negative, and his angiotensin-converting enzyme (ACE) level was elevated. His physical examination showed that he was hemodynamically stable and afebrile and that his overall health status was good. His musculoskeletal examination revealed a raised fistulous erythematous lesion with serous bloody secretion. He had no erythema or joint effusion. In an examination of his skin, no erythematous bullous lesions were found on his lower extremities.

The patient’s blood examination results showed elevated acute-phase reactants (erythrocyte sedimentation rate, C-reactive protein) but no leukocytosis or left shift. His kidney and liver function, electrolytes and clotting were normal. His ACE level was somewhat elevated, and his urine sediment was normal. The results of his serologic tests were negative (hepatitis, HIV, rubella, cytomegalovirus, Epstein-Barr virus, *Toxoplasma*, *Treponema pallidum*, *Borrelia*, *Mycoplasma* and *Chlamydia*). To rule out the possibility of tuberculosis, a Mantoux test (negative) and serial urine cultures with auramine staining (negative) were ordered. His high-resolution CT scan was compatible with stage I sarcoidosis (bilateral hilar and mediastinal adenopathies).

**Fig. 1:**
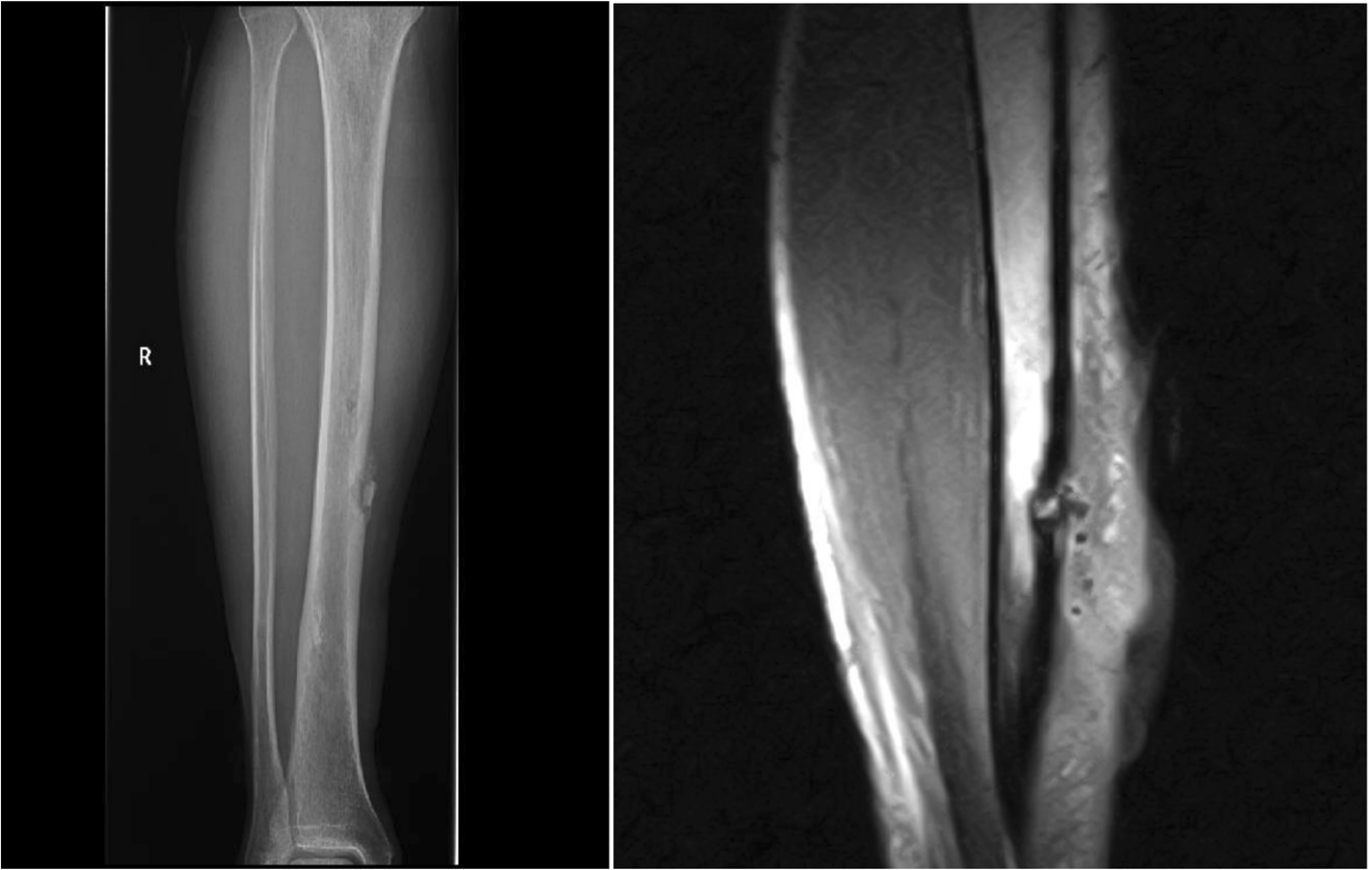
A lesion resembling osteomyelitis on a computed tomographic scan of the left lower limb

**Fig. 2:**
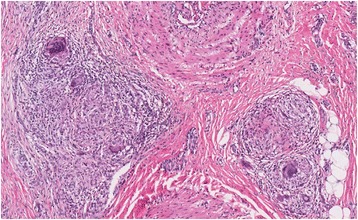
Epithelioid granuloma with multi-nucleated giant cells without necrosis or acid-fast rod microorganisms (AFB) microorganisms examined using Ziehl-Neelsen and periodic acid–Schiff staining techniques

The patient was also being followed in the internal medicine department for a possible infection after a lesion resembling osteomyelitis was observed on a CT scan of the left lower limb (Fig. 1). A biopsy was taken, the result of which was compatible with foreign body granuloma. Culture results were negative, and no malignant cells were found, although *Pseudomonas* and *Corynebacterium* spp. were isolated in the fistulous secretion. Antibiotics were prescribed for 6 months. With no improvement, the patient was referred to the rheumatology department. A second pre-tibial skin biopsy showed a giant-cell granuloma. Ziehl-Neelsen and periodic acid–Schiff staining results were negative (Fig. 2).

At the same time that the lesion on the patient’s left leg worsened, his eye symptoms returned, so he was referred by the ophthalmology department to be assessed for immunosuppressant treatment. Despite improvement with topical and oral corticosteroid therapy, his symptoms persisted.

Treatment was started with prednisone, with the dose being gradually increased from 15mg/day to 30mg/day. The patient showed a very good clinical response. He remained afebrile, his malleolar swelling decreased and his fistulous lesion progressively disappeared. Treatment with 15mg methotrexate was immediately prescribed as a measure to avoid corticosteroid use to a certain extent and to reduce the dose by up to 7.5mg every 2 weeks. The patient had a successful recovery.

The patient was subsequently followed in the rheumatology, trauma and internal medicine departments, where he was found to be asymptomatic with no further attacks of uveitis. The lesion did not recur.

## Discussion

Sarcoidosis is a multi-systemic granulomatous disease of unknown etiology that particularly affects young people. Its most common symptoms are bilateral hilar adenopathies, pulmonary infiltrates and skin and eye lesions. It is also characterized by the formation of non-caseating granulomas [13–15].

When bone is affected, the patient is normally asymptomatic. The presentation of sarcoidosis is described as lytic and cystic lesions on the fingers and toes (42%) that do not generally require treatment. However, soft tissues can be affected, with formation of fistulas on the skin that require curettage and corticosteroid therapy or chloroquine, which generally work very well with cutaneous sarcoidosis. The incidence of these lesions is 1–13%, and bone lesions due to sarcoid granulomas are diagnosed by biopsy [3, 13–16].

Approximately 38% of all cases of erythema nodosum are related to sarcoidosis in Löfgren syndrome, and they occur with skin lesions such as plaques, nodules and scars [17–19]. When the eyes are affected, the cases are usually bilateral, recurrent or chronic and secondary and requires ruling out other systemic causes.

In 50−70% of sarcoidosis cases related to other sites, the eyes are also involved and the eye condition often becomes chronic and recurring [20–22].

ACE values are elevated in 88% of patients with active sarcoidosis, but using these levels to diagnose the disease can be misleading because approximately 10% of results are false positives and about 40% are false negatives. However, measuring the ACE level is useful to monitor disease progression [15].

The cornerstone treatment is based on corticosteroids with gradual dose decreases until the disease is controlled. If the patient’s sarcoidosis is still active, immunosuppressive agents followed by second-line treatments such as methotrexate or azathioprine, as well as biologic agents such as infliximab, can be used for several clinical manifestations, although the evidence for these is based on small studies, so more extensive studies are needed to establish use of these treatments [10, 14, 23].

Four other scar sarcoidosis clinical cases have been reported to date. Scar sarcoidosis was found in all of them. This is an uncommon but specific manifestation of sarcoidosis. One of the patients developed scar sarcoidosis on his right finger [24]. Another patient, who had Löfgren syndrome, developed sarcoidosis after a forehead scar [25]. In two cases, sarcoidosis occurred after a herpes zoster scar. One occurred with a disseminated scar that predicted pulmonary involvement [26].

## Conclusions

To the best of our knowledge, this is the first case of tibia sarcoidosis scar reported in the literature. Sarcoidosis generally responds well to corticosteroid therapy, but it is not self-limiting and often also requires the use of an immunosuppressant to avoid corticosteroids and to achieve remission without recurrence. When the eyes are involved, it is often related to other sarcoidosis symptoms. If the disease is not controlled, these symptoms will not be controlled either. Sarcoidosis is usually treated with methotrexate, cyclosporine, azathioprine or cyclophosphamide. New biologic therapies have also been used, with infliximab producing the best response, although the number of studies on the subject is limited [23, 27, 28].

## Consent

Written informed consent was obtained from the patient for publication of this case report and any accompanying images. A copy of the written consent is available for review by the Editor-in-Chief of this journal.

## Abbreviations

ACE: angiotensin-converting enzyme
CT: computed tomographic
